# Enhanced recovery after surgery—ERAS—principles, practice and feasibility in the elderly

**DOI:** 10.1007/s40520-018-0905-1

**Published:** 2018-02-16

**Authors:** Olle Ljungqvist, Martin Hubner

**Affiliations:** 1 Department of Surgery, Faculty of Medicine and Health, School of Health and Medical Sciences, Örebro University, SE-701 85 Örebro, Sweden; 2 Department of Visceral Surgery, CHUV, Lausanne, Switzerland

**Keywords:** Surgery, Stress, Recovery, Elderly

## Abstract

This is a short overview of the principles of a novel development in surgery called enhanced recovery after surgery (ERAS) programs. This is an evidence-based approach to perioperative care that has shown to reduce complications and recovery time by 30–50%. The main mechanism is reduction of the stress reactions to the operation. These principles have been shown to be particularly well suited for the compromised patient and hence very good for the elderly people who often have co-morbidities and run a higher risk of complications.

## Introduction

Enhanced recovery after surgery (ERAS) is a multi-professional and multidisciplinary approach to the care of the surgical patient. The multi-modal approach to recovery was first outlined by a Danish surgeon, Henrik Kehlet in 1995 for colonic resections [1]. This later developed into what is known as ERAS [2]. The general processes were developed for colonic surgery but have now been adopted to most major operations and even medium size surgeries.

The ERAS protocols have a series of evidence-based care elements that all support recovery by reducing the bodily stress reactions caused by injury. In a very general sense, the endocrine metabolic responses can be reduced from what is seen after a major abdominal open operation to what is developing after a small laparoscopic operation [3]. Similarly, the inflammatory responses are minimized by other treatments shown to have beneficial effects on the recovery. These reductions in the stress responses are of particular importance for the vulnerable patient with co-morbidities, who is often also frail and elderly. This paper gives a short overview of the concepts and reviews the current knowledge of the application to the older patient.

There are three main pillars upon which ERAS lies; evidence-based perioperative care processes, multi-modal and multi-professional teamwork, and continuous audit.

### Evidence-based care

**Fig. 1:**
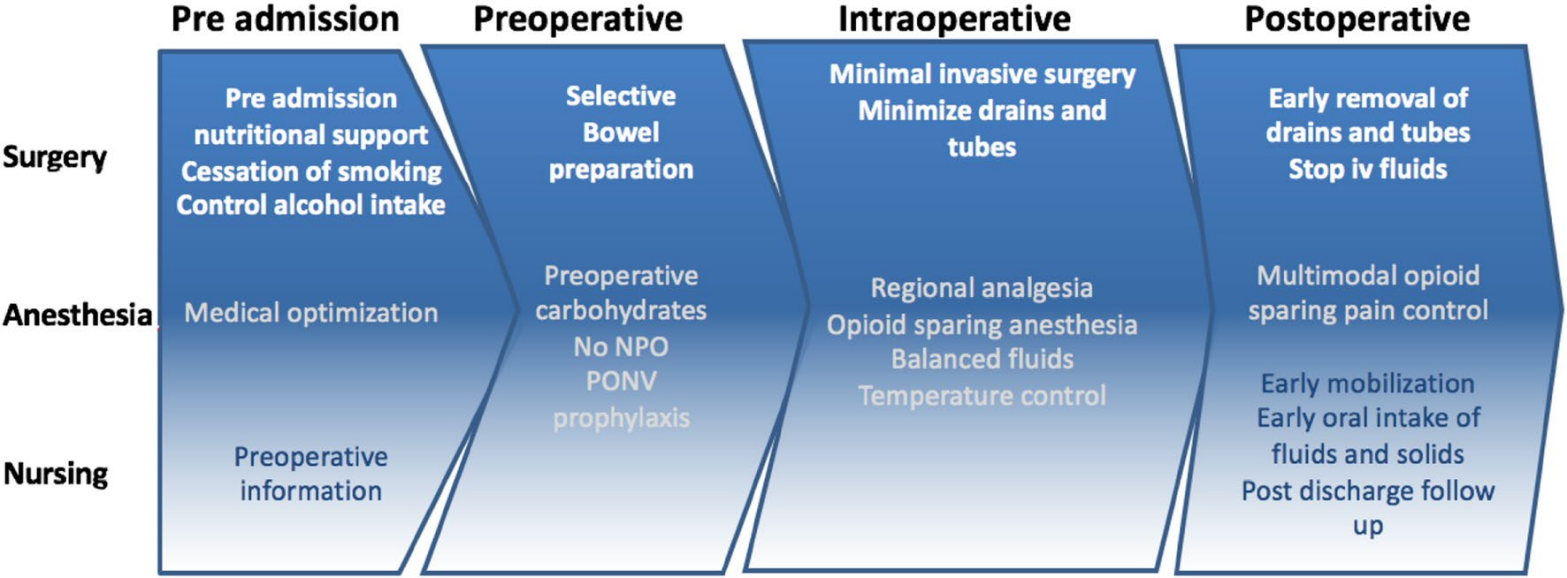
ERAS flowchart. Multi-professional and multidisciplinary approach to management of the patient journey. Modified from Ljungqvist et al., JAMA Surgery 2017. Used by permission © O Ljungqvist

ERAS care protocols are based on the evidence available in the literature and assemble several care elements that all have proven to be supporting recovery and/or avoiding complications after major operations (Fig. 1). These elements form a care plan or pathway that is introduced as the basic standard of care in a unit by engaging everyone involved in the care of these patients. The ERAS® Society, an international non-for profit, multi-professional and multidisciplinary medical society has gathered expertize from around the world to review the literature and publish guidelines for many major surgical procedures in different specialties, all available for free (http://www.erassociety.org).

### The ERAS team

Most hospitals around the world are organized in departments and sometimes sections or units within these departments (outpatient clinic, wards in a department of surgery). In each one of these units professionals are busy managing their day to day work. Very seldom do they have time to see what happens to the patient in the next unit that the patient goes to, nor do they have time to get any insights into what the patient has been given as treatments or preparations in their earlier parts of their journey through care. This means that very seldom is there anybody who knows the details of which treatment the patients receive, and in most countries, there are no national quality registries or other forms of regular independent audit of the care that is provided in any given unit. This means that most often, doctors and nurses do not know the results of their work, and have very little insights into how well they compare with other, nor what may understand that it is the choices they make in their treatments that may cause their patients problems. The focus is often in the short term when the patient is under the care of a caregiver in one unit, and not what that treatment may do for the patient when they arrive in the next unit. A simple example is the management of hypotension during anesthesia. The traditional way is to give fluids. This often resulted in substantial overload of water and salt and the patient gaining weight causing edemas in the GI tract, which in turn causes postoperative ileus. The problem arises several hours after the treatment has been given and the care giver responsible never sees the problem. Nor does the nurse on the ward who faces the problem realize that if the patient had been given less fluids and vasoactive drugs instead to manage the blood pressure during surgery and anesthesia, the problem would never have arisen.

The solution to avoid these problems is to train a team of doctors and nurses to work together, a local ERAS team. The team should have members from each of the units that are involved in the care of the patient; outpatient unit, preoperative site, the OR team including anesthesia, the postoperative recovery unit, intensive care and the wards. This team is trained to run regular meetings, often weekly or bi-weekly. During these meetings, protocols that align and that are based on guidelines are being adopted and applied locally.

### Audit

A very useful tool is an audit tool that allows for continuous data-driven management [4]. Such audit systems will give everyone in the team insights into what is ongoing in their hospital, and allows them to also communicate to all their colleagues about this and this facilitates changes in the right direction. Often it is possible to understand why certain problems are common by checking what was done to the patients with the given complication. This helps direct the team to efforts to reduce the problem while also keeping track that relevant changes in the care are taking place.

### General outcomes in ERAS

Already in 1995, Henrik Kehlet published the ground-breaking paper in the Lancet describing eight older frail patients undergoing elective colonic resections for cancer, feeling fine and being discharged on the second postoperative day [1]. At that time, (and still today in many countries) the length of stay for these patients is 12–15 days. Behind these remarkable data was the so-called Fast Track program, a multi-modal approach to recovery. Later studies in larger series from his unit confirmed these findings for a more general population. While these early studies showed benefits for general recovery, it took until 2010 before it could be shown in meta-analysis that not only length of stay was improved, but that these ERAS programs also caused a marked reduction in complications [5]. Later and larger studies showed that it was mainly the medical complications that were reduced in colorectal ERAS patients; cardiovascular, pulmonary and infectious complications, while surgical complications have so far not shown the same clear advantage from ERAS [6]. Studies in patients with diabetes undergoing hip and knee surgery report that employing ERAS protocols reduce or even abolish the additional risk for complications otherwise associated with operting patients with this diesase [7].

### ERAS in the older patients

There is clear evidence that older and frail patients run a higher risk for complications following major abdominal surgery (British Geriatrics Society). For this reason, it would seem likely that ERAS protocols should have a positive impact on outcomes specifically for this patient group. By minimizing of stress reactions, ERAS protocols should avoid stressing vulnerable and compromised organs.

When discussing risks in surgery for older persons one question is defining older persons. The literature varies from cut offs at above 70 to older. Obviously, there will be large variations in the presence of co-morbidities in this patient group, but that is how the data are being presented in most studies that are available. As the literature is sparse so far, there are shortcomings in the knowledge limited by the types of surgeries that have been studied and the different reactions and protocols that are in place. The protocol for recovery after a hip or knee replacement is going to be different from a colorectal resection. The protocol in place will also be different for that same reason and hence knowledge from one operation to another may not always be valid.

In the following, the available literature in patients undergoing ERAS at the age of 70 years or older will be presented.

In studies of patients above 70, an Italian database study of patients treated according to ERAS principles during colorectal surgery showed that the older patients had more complications overall, but not more serious complications. The older patients mobilized slightly slower self-care, but only if this was found only for the higher risk patients [8]. A Swiss study of similar kind reported more (10 vs 4%) cardiovascular complications in the older patients, while all other complications were the same [9]. A similar cut off for gastric resection from Korea showed a half day longer stay in the older patients, but no difference in complication rates that were generally low [10]. A UK study reported 2 days longer stay for patients over 75 compared to those below that age following colorectal surgery in ERAS [11]. There was no difference in complications.

A Polish study compared patients over 80 with those below 55 undergoing ERAS and laparoscopic surgery [12]. In this study, they determined compliance with the ERAS society guideline recommendations and showed it to be equally high (85%) in both groups. With this care protocol, there was no difference in length of stay, complications or recovery of different functions between groups. The only difference was a need for more rescue opioids in the elderly.

A Danish study in Fast Track hip and knee of patients above 85 years (median 87) showed that most patients were fit to leave the hospital in 3 days [13]. The authors also identified the need for anemia management both pre- and postoperatively in this patient group.

There are two randomized trials comparing ERAS with more traditional care pathways. One study from China recruited patients over 70 undergoing laparoscopic colorectal surgery [14]. With approximately 40 patients in each group, the authors reported fewer complications (5 vs 21%), and faster return of functions as well as shorter hospital stay by 1.5 days down to 5.5 days. Another RCT from China in a similar patient group but this time undergoing open colorectal surgery also found better outcomes with ERAS [15]. Cardiac and pulmonary complications as well as urinary infections were less common in the ERAS group. Delirium was reduced from 13 to 4% and length of stay reduced from 13 to 9 days.

Another aspect of the introduction of ERAS protocol is how they may affect the risks of co-morbidities. One very good example is the studies from Denmark in hip and knee replacement. Information from large database of consecutive patients has shown that the Fast Track/ERAS program used allowed patients with diabetes treated by nutrition or oral medication to reduce the risk to that of a healthy person, while a slightly elevated risk of complications remain for patients on insulin [7]. Another interesting treatment modal that is emerging is the concept of prehabilitation. This is a program where patients take on physical training and take additional whey protein supplementation to strengthen their mobility [16]. Studies are emerging indicating that it is the most vulnerable and fragile with the lowest physical capacity that gains the most from these programs. This may develop into an import additional preparation for the older and fragile patient.

In summary, available data all point in the direction that ERAS protocols are beneficial for older and fragile patients. The stress-reducing program of ERAS serves these patients especially well by reducing complications and supporting the recovery. However, the data are still sparse and more research is needed for this group and for many different surgeries.
